# Effectiveness of behavioral interventions and behavior change techniques for reducing soft drink intake in disadvantaged adolescents: A systematic review and meta‐analysis

**DOI:** 10.1002/osp4.452

**Published:** 2020-10-08

**Authors:** S. S. Shagiwal, E. Groenestein, A. Schop‐Etman, J. Jongerling, J. van der Waal, G. Noordzij, S. Denktas

**Affiliations:** ^1^ Department of Psychology, Education and Child Studies, Erasmus School of Social and Behavioral Sciences Erasmus University Rotterdam The Netherlands; ^2^ Department of Public Administration and Sociology, Erasmus School of Social and Behavioral Sciences Erasmus University Rotterdam The Netherlands; ^3^ Erasmus University College Erasmus University Rotterdam The Netherlands

**Keywords:** behavior change interventions, health inequalities, obesity, sugar‐sweetened beverages

## Abstract

Reducing sugar‐sweetened beverage (SSB) intake is an important dietary target, especially among socioeconomically disadvantaged ethnic minority adolescents. This review and meta‐analysis evaluated the effectiveness of behavioural interventions aiming to reduce SSB intake in socioeconomically disadvantaged ethnic minority adolescents and examined which behaviour change techniques (BCTs) were most effective. A systematic search was conducted using the PRISMA criteria. Quality assessments were done using the Cochrane criteria. In a narrative synthesis, studies were divided into effective and non‐effective, and relative effectiveness ratios of individual BCTs were calculated. Pooled standardized mean differences (SMDs) and their 95% confidence intervals were estimated with random‐effects models using cluster robust methods. Twenty‐two studies were included in the qualitative synthesis. A meta‐analysis (*n* = 19) revealed no significant between‐group differences in reduction of SSB intake. Five self‐regulatory BCTs had an effectiveness ratio >50%: feedback, goal‐setting, action planning, self‐monitoring and problem‐solving/barrier identification. The risk of bias assessments were judged to be moderate to high risk for randomized controlled trials (RCTs) studies and low to moderate for pre–post studies. There was no indication of publication bias. In conclusion, self‐regulatory BCTs may be effective components to change SSB behaviour. However, high‐quality research is needed to evaluate the effectiveness of behavioural interventions and identify BCTs effective for reducing SSB intake among disadvantaged adolescents with ethnic minority backgrounds.

## 1 INTRODUCTION

Adolescent overweight and obesity is a major public health priority because of its link to adverse short‐ and long‐term outcomes.^1^ Compounding the seriousness of the adolescent overweight and obesity trend are stark disparities by ethnic minority status (i.e., residents with immigrant origins), with minority adolescents with low socioeconomic status (SES, e.g., parental education, occupation status or household income) being disproportionately affected by obesity.^2^ In developed countries, higher overweight and obesity rates are reported among socioeconomically disadvantaged ethnic minority adolescents than in advantaged ethnic majority adolescents.^3^ Given these findings, the increased risk of morbidities during adolescence and adulthood and the anticipated increase in socioeconomic disadvantage and ethnic diversity, effective prevention efforts are warranted to circumvent obesity‐related burden in these adolescents.

Disparities in sugar‐sweetened beverage (SSB) intake, defined as nondiet, non‐alcoholic and non‐dairy cold or warm drink with added sugars including sodas, diet drinks, fruit juices (excluding 100% fruit juice), flavoured juice drinks, sports drinks, energy drinks and sweetened tea and coffee,^4^ have been suggested to be a contributing factor to the obesity epidemic.^5^ A high intake of SSBs has been shown to be associated with adolescent obesity.^6^ While health behaviors such as SSB intake are ultimately based on individual decisions, socio‐ecological models suggest that such behaviors are shaped and influenced by the social and physical contexts.^7^ Therefore, it is widely acknowledged that the mechanisms underlying the observed differences in SSB intake are multifactorial, involving a complex web of interacting individual, sociocultural, and environmental factors.^8^

Ethnic patterning in SSB intake might be explained by the multiple socioeconomic disadvantages that underserved adolescents face, including growing up in low‐educated households, interacting with peers who encourage obesogenic‐related dietary behaviors and living in segregated deprived areas characterized by a high density of obesogenic food outlets.^9^ The inverse relationship between individual, family and area‐level indicators of SES is well established; with disadvantaged adolescents exhibiting poor dietary behaviors than their advantaged ethnic majority peers.^10^ These findings are attributed to the fact that these adolescents are less health‐conscious, have limited nutritional knowledge due to individual or parental education level, and lack financial resources, which can restrict food choices.^11^

Within the social and physical home environments, parental SSB intake, permissive food‐related parenting styles and home accessibility and availability of SSBs have been linked to a high SSB intake among disadvantaged minority adolescents.^12^ Next to the family context, peers can also influence individual SSB intake. Seen from the group norm and socialization perspectives,^13^, ^14^ peers help to create norms concerning a behavior (e.g., what SSB behaviors are appropriate and socially acceptable). Given their developmental stage, peer influences are influential during middle adolescence (14–16 years of age).^7^ Because this developmental stage is marked by a heightened need for peer approval and identity, it is assumed that peers could influence individual SSB intake possibly via negative peer modelling and conformity to pro‐SSB social norms.^7^

Underserved adolescents, residing or attending schools located in low‐income, high ethnic minority and socially deprived neighborhoods, are found to have fewer health‐promoting social norms and are often characterized by a high density of obesogenic food environments.^15^, ^16^ Neighborhood‐level variables such as SSB availability, accessibility, affordability and disproportionate advertising and targeted marketing of SSBs in deprived areas have been linked to higher individual SSB intake and purchasing.^15^, ^16^, ^17^

Because adolescents spend majority of their time in schools, school correlates of SSB intake have also been explored. School food environment and the obesogenic environment around schools in deprived neighborhoods have been shown to influence individual SSB behavior through increased availability, affordability, convenience and in‐store price promotion of SSBs.^15^, ^16^, ^18^

Public health initiatives such as education campaigns, nutrition guidelines and school policies restricting sales of SSBs have been undertaken to reduce SSB intake.^19^ Although important, initiatives that rely on educating or informing individuals are often not sufficient enough to engender behavior change,^20^ given that humans are not rational and biased when it comes to their behaviors and recognizing the mindless nature of dietary behaviors.^21^ Additionally, these initiatives address only one context and are often not theory driven. As a result, they are more likely to lead to *intervention‐generated inequalities*,^22^ because evidence shows that disadvantaged groups with poor literacy skills are less receptive to interventions that rely on educating or informing individuals.^20^, ^23^ Recently, there has been a growing appreciation for using theory‐driven behavioral interventions defined as a coordinated set of activities designed to change specified behaviour patterns.^24^ Theory‐based interventions not only provide a framework for designing interventions but also facilitate an understanding of the underlying mechanisms that drive behavior change.^24^ Additionally, they can be used as a guide to identify behaviour change techniques (BCTs) described as the ‘active ingredients of an intervention designed to change behavior’.^25^ To date, more than 93 BCTs have been identified.^26^

Previous systematic reviews and meta‐analyses of behavioral interventions targeting adolescent SSB intake suggest that although these interventions lead to small reductions in SSB intake, more evidence regarding their effectiveness is needed.^4^, ^27^, ^28^, ^29^ Some limitations of existing reviews and meta‐analyses are that they have included adolescents from the general population or have examined the effectiveness of behavioral interventions within specific settings such as preventive eHealth interventions in school settings.^27^ Thus far, there has been little research analysing the effectiveness of behavioral interventions and BCTs on reducing SSB intake in disadvantaged minority adolescents. In fact, a systematic review examining the impact of behavioral interventions of different health behaviors concluded that there was a ‘widespread paucity’ of evidence about the effectiveness of changing health behaviors (including dietary behaviors) in underserved groups. In light of these findings, the aims of this systematic review and meta‐analysis were twofold: (a) to review existing behavioral interventions aimed at reducing SSB intake among 12‐ to 18‐year‐old disadvantaged minority adolescents and (b) to identify active BCTs effective in reducing SSB intake.

## 2 METHODS

### 2.1 Search methodology, data screening, extraction and methodological assessments

The current systematic review and meta‐analysis is registered in PROSPERO (the International Prospective Register of Systematic Review, registration ID: CRD42018109645) and was conducted according to the PRISMA statement (available upon request).^30^ A library specialist performed a systematic search with no date restrictions in MEDLINE (OVID), EMBASE, Web of Science, Cochrane Central Register of Controlled Trials and Google Scholar from inception to July 2018 (available upon request), for randomized controlled trials (RCTs), non‐RCTs, quasi‐experimental, pre–post studies, published in English, of behavioural interventions for reducing SSB intake, measured either subjectively or objectively at baseline and at post‐intervention, in 12‐ to 18‐year‐old disadvantaged ethnic minority adolescents (Table 1).

**TABLE 1:** PICO framework for study eligibility

Parameter	Inclusion criteria
Participants	Nonclinical socioeconomicallly disadvantaged ethnic minority adolescents aged 12–18 years.
Intervention	Behavioral interventions:
	‐ Targeting a reduction in SSB intake as one of the main intervention targets
	‐ Delivered in any setting (e.g., home, school and communities)
	‐ Using behavior change techniques (BCTs) designed to reduce SSB intake
Comparison	Control groups with no intervention (e.g., usual care, active, waitlist)
Outcomes	Outcome measure is SSB defined as a non‐diet, non‐alcoholic and non‐dairy cold or warm drink, carbonated or still, with added sugars. Including fruit drinks. Nectars with <100% fruit. Sports and energy drinks, sweetened tea and coffee.^4^
	Primary outcome measures
	‐ Reduction in SSB consumption quantified as difference between pre‐ and post‐intervention and where possible follow up. Quantity of SSB consumed (ml of SSB consumed per day per week) and frequency of SSB consumed (e.g., percentage of participants consuming a given quantity of SSB), purchases of SSB, energy intake from SSB (e.g., total energy intake in kcal/day or per person) will be included
	‐ Studies using different types of SSBs. the outcomes could be reported separately (e.g., soft drinks and sports drinks) or collectively (i.e., all types of SSBs)
	Secondary outcome measures
	‐ Subjective changes in knowledge/attitude/beliefs related to SSB intake
Study design	Randomized controlled trials (RCTs) (e.g., cRCTs). Quasi‐experimental (e.g., with pre–post designs) and one‐group pre–post studies. With baseline. Post‐intervention and where possible. Follow‐up data.
**Exclusion criteria**	
The following studies were excluded:	
‐ Animal studies	
‐ Studies conducted in adults	
‐ Studies reporting only baseline data	
‐ Case–control. Cohort. cross‐sectional and longitudinal studies	
‐ Systematic reviews and meta‐analyses (to maintain consistency and because it is difficult to interpret results from previous meta‐analyses pooling estimates from RCTs using different control groups. We decided to exclude them from the current study	
‐ Meetings/congress reports	

Abbreviation: PICO, participant, intervention, control/comparison and outcomes; RCT, randomized controlled trials; SSB, sugar‐sweetened beverage.

To identify studies missed by the electronic search, one reviewer (S. S.) screened the reference lists of selected studies and of relevant reviews to retrieve studies that met the eligibility criteria. Where information was needed, authors were contacted to provide the missing data. Studies identified through the electronic search were exported to Endnote for removal of duplicates. Both reviewers (S. S. and E. G.), independently screened the abstracts, titles and full texts of retrieved studies. Interrater agreement of record screening and inclusion of studies between reviewers was 98%. Disagreements on eligibility were resolved through discussion. One reviewer (S. S.) extracted data of all included studies using an adapted Cochrane spreadsheet form.

Two reviewers (S. S. and E. G.) assessed the risk of bias of RCTs using the Cochrane risk of bias tool (RoB)^31^ in Review Manager (Version 5.3 Cochrane, London, UK). One reviewer (S. S.) assessed the quality of non‐RCTs using the Newcastle–Ottawa Scale (NOS).^32^ For the latter risk of bias assessment, a maximum of nine points was allocated for the least risk of bias in three domains: (a) selection of bias (max 4 points); (b) comparability of the study (max 2 points); and (c) ascertainment of exposure and outcomes (3 points). The scores were subsequently categorized as: high (0–3 points), moderate (4–6 points) or low (7–9 points).

### 2.2 Coding of BCTs

The BCT taxonomy v1 (BCTTv1)^26^ was used to extract BCTs. One reviewer (S. S.) completed an online BCT course prior to coding of BCTs. A BCT was only coded if there was clear evidence of inclusion. Interventions targeting multiple behaviors (e.g., physical activity and dietary behaviors) where possible, BCTs related to SSB intake were coded. If this was not possible, only BCTs related to healthy eating were coded. BCTs present in intervention arms were recorded only. Studies using multiple intervention arms, the BCTs were extracted from each active intervention arm.

### 2.3 Statistical analysis

The primary outcome was a reduction in SSB intake. Due to the variability in the measures used for SSB intake, a hierarchy was developed whereby the most empirically supported method in each study was preferred. Daily SSB servings were prioritized above volumetric measures or SSB consumed in the past month, because the latter would be prone to the wrong estimation and recall bias, respectively. Effect estimates, reported either separately for each type of SSB (e.g., soda and fruit juice) or collectively (all SSB types categorized into one group) were extracted.^33^, ^34^, ^35^, ^36^ Only studies that reported SSB intake at pre‐intervention and immediately at post‐intervention were included because most studies did not assess long‐term effectiveness. In studies with multiple intervention arms, each intervention arm was compared with the control arm at each time point.^37^

In total, 15 studies reported mean SSB intake,^35^, ^36^, ^37^, ^38^, ^39^, ^40^, ^41^, ^42^, ^43^, ^44^, ^45^, ^46^, ^47^, ^48^, ^49^ and four studies reported proportions of participants consuming a given quantity of SSBs.^33^, ^34^, ^50^, ^51^ Three studies were excluded because the change in SSB intake from baseline was not reported.^52^, ^53^, ^54^ To combine different measures of SSB in one analysis, dichotomous measures were transformed using the Freeman–Tukey double arcsine transformation (i.e., transforming the sampling distribution to a stable normal distribution).^55^ Transformed scores were subsequently used to calculate standardized mean differences (SMDs) between groups. For continuous variables, SMDs were calculated from the raw (untransformed) scores. Meta‐analysis was conducted to assess changes in reported SSB intake at pre‐ and at post‐intervention, and any changes in effect estimates from pre‐ to post‐intervention. For continuous SSB outcomes, means, standard deviations (*SD*s) and sample sizes were extracted for each time point from each group of each study. In cases where other summary statistics were reported (e.g., interquartile range, standard errors and confidence intervals [CIs]), these values were used to compute the missing *SD*s, as described by the Cochrane guidelines.^56^

For each study, the difference in SSB intake at each time point between groups was estimated as the mean difference (mean intervention group at pretest—mean control group at pretest/pooled SD of both groups at pretest). To standardize the effect estimates, computed SMDs were estimated using Hedges' *g* statistics.^56^ SMD effect estimates of 0.3, 0.5 and 0.8 were considered small, medium and large, respectively.^57^ Pooled SMDs with their corresponding 95% CIs were estimated using random‐effects models. These models were used because by nature, behavioral interventions are considerable heterogeneous in terms of intervention features. To account for multiple measures per study, a series of multilevel models were investigated with nested random effects top level being the study, then treatment type and time period using *REML* estimation. Cluster robust meta‐analysis was performed to account for the dependency between multiple measures extracted from the same study.^58^

Heterogeneity between studies was assessed using Cochran's *Q* test and *I* ^2^ statistic.^59^ A significant *Q* test (*p* < 0.05) was suggestive of heterogeneity while *I* ^2^ estimates of 50%–75% and >75% were considered substantial to considerable heterogeneity, respectively.^59^

Subgroup analyses were not feasible given that a minimum of 10 studies is required for a meaningful interpretation.^59^ Instead, exploratory analyses were performed to examine the impact of potential moderators: study design (RCTs, non‐RCTs), intervention type (educational/behavioral, environmental), intervention component (single, multicomponent) and total number of BCTs used. Moderator levels with sample size smaller than two, that is, gender (female or male), use of theoretical framework (yes or no), type of theory used (behavioural or non‐behavioural), setting (school‐based or other), SSB measurement tool (self‐reported or objective) and control group (yes or no) were excluded from the analyses. Intervention duration was excluded as a potential moderator because most of included studies only assessed short‐term effects.

Publication bias was assessed by observation of funnel plots and using Eggers regression asymmetry test.^60^, ^61^ An asymmetric plot and *p* < 0.05 were considered to be suggestive of publication bias.

A sensitivity analysis was performed using fixed effects instead of random effects in the main meta‐analysis. All statistical analyses were conducted in R (v3.5.1, Vienna Austria), using the *metafor and robust packages*.

### 2.4 Narrative synthesis

Owning to the heterogeneity of BCTs included, a narrative synthesis was performed to identify most commonly used BCTs and types of BCTs associated with intervention effectiveness. Using the approach as Gardner et al.,^62^ studies were divided into ‘very effective’, ‘quite effective’ and ‘non‐effective’. Interventions were deemed ‘very effective’ and ‘quite effective’ if significant *between*‐group and *within*‐group differences in SSB intake were reported, respectively. An intervention was coded as ‘non‐effective’ if no significant *within*‐ or *between*‐group differences in SSB intake were reported. In line with other reviews^63^, ^64^ to identify BCTs associated with effective interventions, a percentage effectiveness ratio was calculated, whereby the number of times a BCT was a component of ‘very effective’ and ‘quite effective’ interventions was divided by the number of times a BCT was a component of all interventions. To avoid inflation of results from BCTs used on a few occasions, BCTs that were identified in a minimum of five studies were included in the analysis.^63^, ^64^

## 3 RESULTS

### 3.1 Study selection and characteristics

**FIGURE 1:**
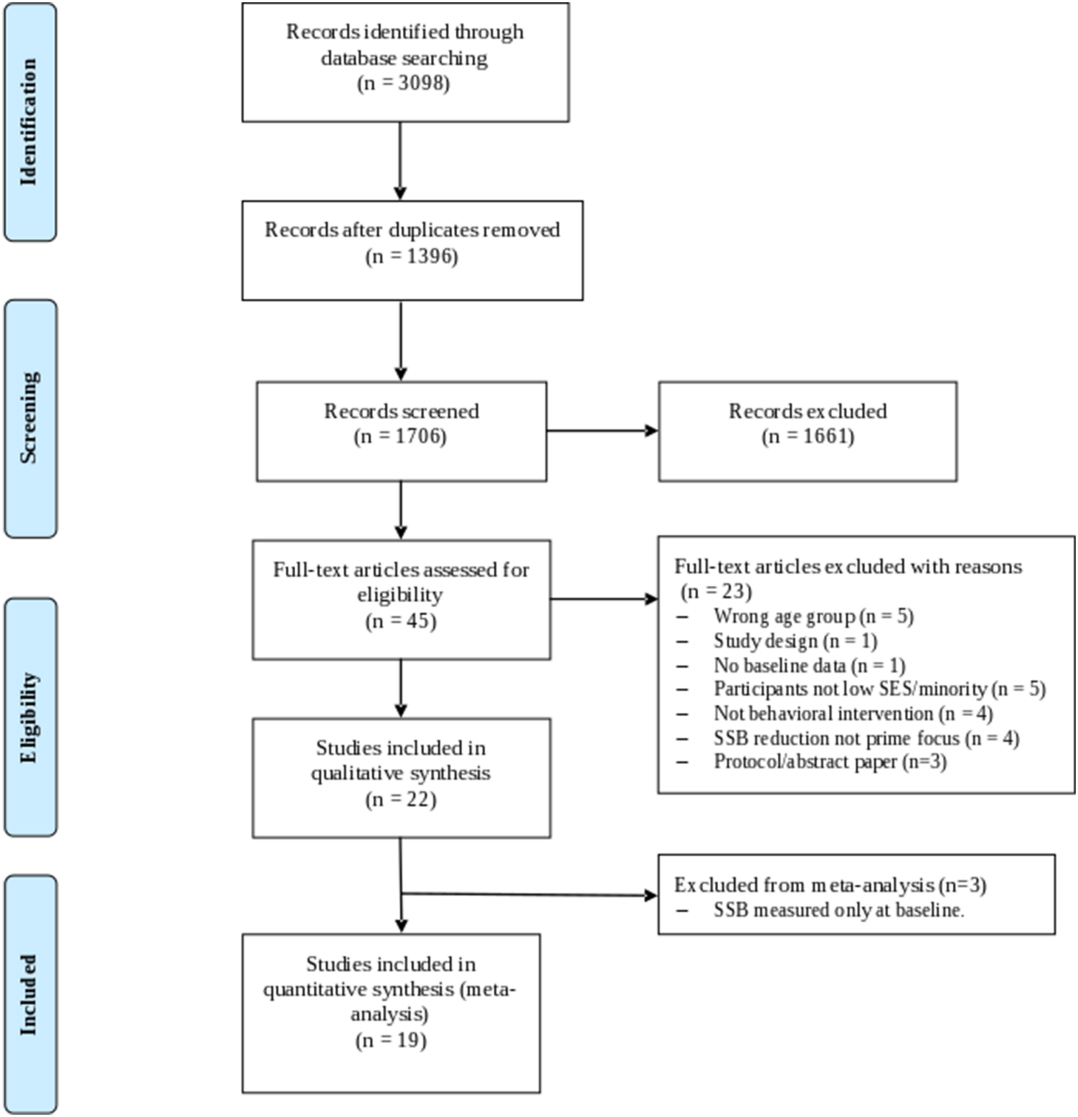
Study flowchart. SSB, sugar‐sweetened beverage

A total of 3,098 studies were identified in the initial search of which 1,396 duplicates were removed. After screening the titles and abstracts of 1,706 studies, 45 studies were selected for full‐text screening. At the final stage, 22 studies were included in the qualitative synthesis of which 19 were included in the meta‐analysis^33^, ^34^, ^35^, ^36^, ^37^, ^38^, ^39^, ^40^, ^41^, ^42^, ^43^, ^44^, ^45^, ^46^, ^47^, ^48^, ^49^, ^50^, ^51^ (Figure 1). Three studies were excluded from the meta‐analysis because only baseline SSB intake was reported.^52^, ^53^, ^54^

The characteristics of the included studies are summarized in Table 2. The sample size of the 22 studies ranged from 43 to 4,249, and comprised 19,934 participants with 14,104 individuals allocated to intervention and 5,830 individuals allocated to control arms. The mean age of participants was 13.65 years (*SD* 0.96) in the intervention arms^34^, ^35^, ^36^, ^37^, ^38^, ^40^, ^42^, ^43^, ^44^, ^46^, ^47^, ^48^, ^49^, ^50^ and 13.44 years in the control arms.^34^, ^37^, ^38^, ^40^, ^42^, ^43^, ^44^, ^46^, ^47^, ^50^ Thirteen studies were conducted in ‘healthy’ (i.e., nonclinical group) adolescents,^33^, ^35^, ^36^, ^37^, ^38^, ^39^, ^41^, ^45^, ^49^, ^51^, ^52^, ^53^, ^54^ and nine studies were conducted in a mix of normal weight, overweight or at risk of overweight/obesity adolescents.^34^, ^40^, ^42^, ^43^, ^44^, ^46^, ^47^, ^48^, ^50^ Three studies assessed SES based on individual‐level SES (i.e., education level),^43^, ^47^, ^50^ three based on family‐level SES (i.e., parental education attainment and occupation status),^37^, ^38^, ^54^ two studies based on household‐level SES (i.e., parental income),^39^, ^48^ four studies based on household‐level SES (eligibility for the national free/reduced school lunch programme),^33^, ^41^, ^42^, ^45^ eight studies based on neighborhood‐level SES,^34^, ^35^, ^36^, ^40^, ^49^, ^51^, ^52^, ^53^ and one study based on school‐level SES assessed by the Index of Community Socio‐Educational Advantage (ICSEA).^44^ One study did not report participants' SES backgrounds.^46^ Fifteen studies included ethnic diverse participants: African–American,^52^, ^53^ Latino/Hispanics,^41^ a combination of Black/African–American, Latino/Hispanics, Asian/Pacific, Native American,^33^, ^35^, ^36^, ^42^, ^44^, ^45^, ^48^, ^54^ non‐Western, White and a mixed ‘other’ group.^34^, ^39^, ^46^, ^50^ Six studies mentioned including an ethnic diverse sample; however, participants' ethnic backgrounds were not reported.^37^, ^38^, ^40^, ^43^, ^47^, ^49^ One study assessed ethnicity based on language spoken at home.^51^ Participants' education levels differed. Education levels were categorized as either middle, high or secondary schools, depending on where the study was conducted.

**TABLE 2:** Detailed summary of study characteristics of 22 studies reporting change in SSB intake

Author, year country	Study aims	Study design	Setting provider	Length of intervention	Sample demographics	Intervention type Theory used	Intervention arm Control arm	BCTs used	SSB type SSB assessment	Overall conclusion
Bleich et al. 2014^52^ USA	To examine whether caloric information reduces the quantity, volume and number of calories of SSB purchases among Black adolescents.	Case‐crossover RCT	**Setting:** Corner store‐based **Provider:** Study staff	**intervention:** 2 weeks **Follow up:** 6 weeks follow up	**Population:** Healthy adolescents *N*: 433 boys, 383 girls **Age:** 12–18 years **Education level:** Middle & high schools **SES measure:** area‐level SES, stores in low‐SES neighbourhoods **Ethnicity:** Black/African–American	Environmental **Theory:** NR	**Intervention group:** Caloric signs on SSBs **Control group:** No caloric signs provided	No BCTs	**SSBs:** Soda, fruit drink, sport drink, vitamin water, and ‘hug’ (a fruit drink packaged in 8‐ounce bottles) **Objective:** registered sales data	Significant reduction in SSB intake
Bleich et al. 2012^53^ USA	To examine whether different forms of caloric information reduces the volume of SSB purchases among Black adolescents.	Case‐crossover RCT	**Setting:** Corner store‐based **Provider:** Study staff	**intervention:** 2 weeks **Follow up:** ?	**Population:** Healthy adolescents *N*: 800 boys, 799 girls **Age:** 12–18 years **Education level:** Middle & high schools **SES measure:** area‐level SES, stores in low‐SES neighbourhoods **Ethnicity:** Black/African–American	Environmental **Theory:** NR	**Intervention group:** Caloric signs on SSBs **Control group (inactive control):** Receives no intervention	No BCTs	**SSBs:** Soda, fruit drink, sport drink, vitamin water, and ‘hug’ (a fruit drink packaged in 8‐ounce bottles) **Objective:** registered sales data	Significant reduction in SSB intake
Bogart et al.^33^ 2011 USA	To pilot‐test an intervention aimed to translate school obesity‐prevention policies into practice with peer advocacy of healthy eating and school cafeteria changes.	Quasi‐experimental (pre–post with a control group)	**Setting:** School‐based **Provider:** Students	**intervention:** 5 weeks **Follow up:** 1‐month follow up	**Population:** Healthy adolescents *N*: 200 boys, 200 girls **Mean age:** 13 years (*SD* 0.5) **Education level:** seventh graders, middle schools **SES measure:** household income‐level SES, i.e., NFLP eligibility (77% NFLP eligible) **Ethnicity:** 68% Latino 17% Asian/Pacific 11% Black/African American 2% White 1% Native American 1% Other	Educational/behavioural/environmental **Theory:** Diffusion theory Social cognitive theory Theory of planned behaviour Empowerment theory	**Intervention:** School‐based peer‐led educational programme with: Peer leader component where peers are trained to model and promote healthy behaviours. School food changes in the environment which included improving canteen signage, providing posters explaining and displaying nutritional information. **Control (inactive control):** Receives no intervention	2.2, 4.3, 5.1, 12.1, 13.1	**SSBs:** Soda, sports drinks and noncarbonated fruit drinks **Subjective:** Dietary survey	Significant within‐group reduction in SSB intake.
Collins et al.^34^ 2014 Australia	To test the impact of a school‐based obesity prevention programme targeting adolescent girls of low socioeconomic position on dietary intake and behaviours	Cluster‐RCT	**Setting:** School‐based **Provider:** School staff	**intervention:** 12 months **Follow up:** 12 months follow up	**Population:** Normal (58%) and at risk for OWOB sample *N*: 357 girls **Mean age:** 13.1 years (*SD* 0.44) **Education level:** High schools **SES measure:** area‐level SES, schools in low‐SES neighbourhoods **Ethnicity:** Australian: 153 (86%) European: 18 (10%) Other: 8 (4.6%)	Education/behavioural **Theory:** Social cognitive theory	**Intervention:** Handbook on nutrition and physical activity Three practical nutritional workshops Parental newsletters and text‐messaging **Control (waitlist control)** Receives intervention after intervention period ends	2.3, 3.2, 5.1, 7.1	**SSBs:** Soda, fruit juice drinks and cordial concentrate **Subjective:** validated FFQ	No significant between‐group reduction in SSB intake
Contento et al. 2007^36^ USA	To evaluate the feasibility of a school‐based intervention (C3) on fostering healthful eating, physical activity and healthy weight through enhancing agency and competence among middle school students.	Quasi‐experimental (pretest‐posttest with no control group)	**Setting:** School‐based **Provider:** School staff	**intervention:** 8 weeks	**Population:** Healthy adolescents *N*: NR **Mean age:** 12 years (range 11–13) **Education level:** 7th graders, middle schools **SES measure:** Area‐level SES, schools in low‐SES neighbourhoods **Ethnicity:** 25% African American, 70% Hispanic 5% other	Education/behavioural **Theory:** Extended theory of planned behaviour Self‐regulation theory Theory of planned behaviour	**Intervention:** Inquiry‐based science education consisting of a 24‐session curriculum during which students learns how one's body works, how to set goals and learning skills to achieve goals **Control:** No control group	1.1, 1.2, 1.4, 1.9, 2.3, 5.1, 10.11	**SSBs:** Carbonated/noncarbonated drinks **Subjective:** Validated FFQ	Significant within‐group reduction in SSB intake
Contento et al. 2010^35^ USA	To examine the impact of a curriculum intervention, choice, control, and change (C3), on energy balance‐related behaviours (EBRBs) such as decreasing SSB intake and on potential mediators of the behaviours.	Cluster‐RCT (pre–post with a control group)	**Setting:** School‐based **Provider:** School staff	**intervention:** 1 year	**Population:** Healthy adolescents *N*: 286 boys, 275 girls **Mean age:** 12 years (range 11–13) **Education level:** Middle schools (7th graders) **SES measure:** Household income‐level SES, i.e., NFLP eligibility (83% NFLP eligible) **Ethnicity:** 25% African American 70% Latino 5% others	Educational/behavioural **Theory:** Social cognitive theory Self‐determination theory	**Intervention:** Inquiry‐based education which consists of 24 sessions of 45 min addressing energy balance, educational activities for acting, potential motivational and behaviour change variables to enhance personal agency. **Control (waitlist control):** Receives intervention after intervention period ends	1.1, 1.2, 1.4, 1.9, 2.3, 4.1, 5.1, 10.11	**SSBs:** Soft drinks, fruit drinks, sports drinks, iced tea, drink mixes **Subjective:** validated FFQ	Significant between‐group reduction in SSB intake.
Dubuy et al. 2012^38^ Belgium	To examine the impact of a curriculum intervention, choice, control, and change (C3), on energy balance‐related behaviours (EBRBs) such as decreasing SSB intake and on potential mediators of the behaviours.	Quasi‐experimental (pre–post with a control group)	**Setting:** Schools Football clubs **Provider:** School staff	**intervention:** 4 months	**Population:** Healthy adolescents *N*: 146 boys, 19 girls **Mean age:** 12.6 years (*SD* 1.02) **Education level:** Elementary and secondary schools **SES measure:** Family‐level SES, i.e., parental education level **Ethnicity:** NR	Educational/behavioural **Theory:** Elaboration likelihood model	**Intervention:** Start clinic and end clinic: clinic activities encouraging healthy diet and physical activity (e.g., eating a healthy breakfast) school programme: 4‐month educational programme consisting of classroom activities on healthy diet and physical activity. **Control (usual control):** Receives regular curriculum	1.8, 5.1, 6.1, 9.1	**SSBs:** Soft drinks **Subjective:** Validated FFQ	No significant between‐group reduction in SSB intake.
Ezendam et al. 2012^50^ The Netherlands	To evaluate the short‐ and long‐term effects of FATaintPHAT to prevent excessive weight gain among adolescents aged 12–13 years by improving their dietary behaviours (reducing SSB consumption), reducing sedentary behaviours and increasing PA.	Cluster‐RCT	**Setting:** School‐based **Provider:** School staff	**intervention:** 4 months **Follow up:** 2‐year follow up	**Population:** Normal (75%) and at risk for OWOB sample *N*: 284 boys, 198 girls **Mean age:** 12.7 years (*SD* 0.7) **Education level:** General secondary schools **SES measure:** Individual‐level SES, i.e., education level (64% vocational) **Ethnicity:** 320 Western 165 non‐Western	Educational/behavioural **Theory:** Precaution adoption process model Implementation intentions Theory of planned behaviour	**Intervention:** Educational component: Online computer‐tailored intervention consisting of 8 lessons spread over 10 weeks. Each module consists of information about the behaviour‐health link, assessment of behaviour, tailored feedback on dietary and physical activity behaviours, and option to formulate an implementation intention to prompt specific goal setting and action planning. **Control (usual control)** Receives regular curriculum	1.1, 1.2, 1.4, 2.2, 3.2, 4.1 5.1, 6.2	**SSBs:** Type SSB NR **Subjective:** Validated FFQ	Significant between‐group reduction in SSB intake
Foley et al. 2017^51^ Australia	To assess the impact of the SALSA programme on Year 10 SALSA peer leaders ‘dietary, physical activity and recreational screen time behaviours, and their intentions regarding these energy balance‐related behaviours (EBRBs)	Quasi‐experimental (pre–post with no control group)	**Setting:** School‐based **Provider:** Students	**intervention:** NR	**Population:** Healthy adolescents *N*: 150 boys, 265 girls **Age:** Range 13–16 years **Education level:** High schools **SES measure:** Area‐level SES, schools located in low‐SES neighbourhoods **Ethnicity:** Language spoken at home 70% English 19% Asian 6% Middle Eastern 5% Other	Education/behavioural **Theory:** Social Cognitive Theory Empowerment education approach WHO Health Promoting School Framework	**Intervention:** Peer education intervention: SALSA educators (university students) will train year 10 students as SALSA peer leaders. In teams of four, trained leaders will deliver 4 × 70 min lessons to their young peers in Year 8 using DVD, games and activities on how to make healthy dietary choices and be physically active **Control:** No control group	1.1, 1.3, 1.4, 4.1, 6.1	**SSBs:** Type SSB NR **Subjective:** Validated FFQ	Significant within‐group reduction in SSB intake
French et al. 2011^39^ USA	To evaluate the effects of a family‐based intervention to prevent excess weight gain among a community‐based sample of households (HH)	cluster‐RCT	**Setting:** Household‐based **Provider:** Study staff	**intervention:** 6 months **Follow up:** 6 months	**Population:** Healthy adolescents *N*: 75 boys and girls **Age:** 12–17 years old **Education level:** General secondary schools **SES measure:** Household‐level SES, i.e., parental income (34% ≤ $45,000/year) **Ethnicity:** 21% non‐White	Education/behavioural **Theory:** NR	**Intervention:** Home‐based programme which included 6 monthly: Face‐to‐face group sessions: 2 h group sessions on behavioural education, physical activity and healthy dietary choices, monthly newsletters and 12 home‐based activities and telephone support calls. **Control (inactive control):** Receives no intervention	1.1, 1.2, 1.4, 2.3, 3.2, 6.2, 7.3, 10.3, 12.1, 12.5	**SSBs:** Type SSB NR **Subjective:** Validated FFQ	No significant between‐group reduction in SSB intake.
Haerens et al. 2007^37^ Belgium	To evaluate the effects of a middle‐school healthy eating promotion intervention combining environmental changes and computer‐tailored feedback, with and without an explicit parent involvement component.	Cluster‐RCT	**Setting:** School‐based **Provider:** School staff	**intervention:** 12 months **Follow up:** NR	**Population:** Healthy adolescents *N*: Intervention arm 1: 734 boys, 491 girls Intervention arm 2: 849 boys, 156 girls **Age:** Intervention arm 1 13.04 (*SD* 0.79) intervention arm 2: 13.2 (*SD* 0.87) **Education level:** General secondary schools with technical/vocational training **SES measure:** Family‐level SES, i.e., parental occupation (68% of participants low‐SES) **Ethnic background:** Study mentions including ethnical diverse sample but participants' backgrounds are NR	Education/behavioural/ Environmental **Theory:** Transtheoretical model Theory of planned behaviour	**Intervention:** —Educational component aimed to promote healthy food choices and physical activity engagement ‐environmental changes: increasing the availability of healthy foods and restricting the availability of unhealthy foods by implementing policies. To increase fruit intake, fruits were sold at very low cost/provided free to all 7th and 8th graders. To increase water consumption, schools offered free drinking fountains ‐parental involvement: parents received newsletters on how to create supportive home environments for health behaviours. **Control (inactive control)** Receives no intervention	1.2, 1.4, 2.2, 3.2, 4.1, 5.1, 6.1, 6.2, 12.1	**SSBs:** Soft drinks **Subjective:** Validated FFQ	No significant between‐group reduction in SSB intake.
Lane et al. 2018^40^ USA	To test the feasibility of KidsSmartER intervention on reducing SSB intake among 6th and 7th graders.	Matched‐contact crossover RCT with no control group	**Setting:** School‐based **Provider:** School and study staff	**intervention:** 6 weeks **Follow up:** 3 months follow up	**Population:** Normal (*n* = 12) and at risk for OWOB sample *N*: 17 boys, 26 girls **Mean age:** 11.7 years (*SD* 0.6) **Education level:** Middle schools **SES measure:** area‐level SES, schools located in low‐SES neighbourhoods **Ethnicity:** Racial/ethnic adolescents, backgrounds NR	Education/behavioural **Theory:** Theory of planned behaviour	**Intervention:** School‐based educational curriculum (6‐week 45‐min lesson given during science classes) with different components: Media literacy to encourage obtaining, interpreting and controlling the influence of media messages Public health literacy to obtain, interpret and act on information needed to make decisions benefitting the community. **Control (active control)** Control group received matched‐contact intervention	1.1, 1.2, 3.2, 5.1, 5.3, 6.1, 13.1	**SSBs:** Soda, energy/sports drinks, coffee with cream/sugar, sweet tea, and sweetened fruit juice **Subjective:** Validated FFQ	Significant within‐group reduction in SSB intake.
Majumdar et al. 2013^41^ USA	To evaluate the efficacy of a game on decreasing intake of processed snacks (e.g., chips, candy) and SB among adolescents attending low‐income schools.	Pretest‐posttest‐matched design with a control group	**Setting:** School‐based **Provider:** Game	**intervention:** 1 month	**Population:** Healthy adolescents *N*: boys, 88 girls **Age range:** 11 to >13 years **Education level:** 6th and 7th graders in middle schools **SES measure:** Household‐income based on NSLP eligibility (78% NSLP eligible) **Ethnicity:** 63% Latino/Hispanics	Education/behavioural **Theory:** Social Cognitive Theory Self‐determination theory	**Intervention:** game‐based educational programme consisting of 24 lessons targeting dietary and physical activity behaviours **Control (active control):** Control receives a different game (WhyWille) game which did contain active intervention components	1.1, 1.2, 1.4, 2.3, 4.4, 5.1, 7.1	**SSBs:** Type SSB NR **Subjective:** Validated FFQ	Significant between‐group reduction in SSB intake.
Neumark‐Sztainer et al 2010^42^ USA	To evaluate the impact of a school‐based intervention aimed at preventing weight‐related problems in adolescent girls.	RCT	**Setting:** School‐based **Provider:** Study staff	**intervention:** ? **Follow up:** ?	**Population:** Normal (17%) and at risk for OWOB sample *N*: 356 girls **Mean age:** 17.5 years (*SD* 1.13) **Education level:** High schools **SES measure:** Household‐income based on NSLP eligibility (58% NSLP eligible) **Ethnicity:** 32.4% African–American/Black 27% White 16.5% Hispanic 8% Mixed/other 3.3% American Indian	Education/behavioural **Theory:** Extensive formative research Social cognitive theory Transtheoretical Model	**Intervention:** Educational component consisting of nutrition and social support/self‐empowerment sessions individual counselling sessions, one‐week lunch brunch and parent outreach. **Control (active control)** Receives a different intervention	1.1, 1.2, 3.2, 4.1, 5.1, 8.1, 11.2	**SSBs:** Soda, fruit drinks, sports drinks, sweetened tea and coffee **Subjective:** 24‐h recall	No significant between‐group reduction in SSB intake.
Singh et al. 2009^43^ The Netherlands	To evaluate the efficacy of a multicomponent intervention on reducing SSB intake in both short and long‐term terms among Dutch adolescents.	cluster‐RCT	**Setting:** School‐based **Provider:** Study staff	**intervention:** 8 months **Follow up:** 12 months	**Population:** Normal (71.8%) and at risk for OWOB sample *N*: 295 boys, 337 girls **Mean age:** 12.8 years (*SD* 0.5) **Education level:** Prevocational secondary schools **SES measure:** Individual‐level SES, i.e., education level **Ethnicity:** Author mentions including a diverse ethnic sample but participants backgrounds NR	Education/behavioural/ environmental **Theory:** Intervention mapping	**Intervention:** —Educational programme covering 11 lessons on biology and physical education —Environmental change options such as encouraging schools to offer additional physical education classes and advice for schools on changes in and around school cafeterias **Control (usual control):** Receives regular curriculum	1.2, 1.4, 2.2, 2.3, 3.2, 4.1, 5.1, 6.2, 7.1, 7.3, 8.1, 8.7, 10.3, 12.1, 12.5	**SSBs:** Soft drinks and fruit juices **Subjective:** Validated dietary questionnaire	Significant between‐group reduction in SSB intake.
Smith et al. 2014^44^ Australia	To evaluate the impact of the Active Teen Leaders Avoiding Screen‐time (ATLAS) intervention for adolescent boys, an obesity prevention intervention using smartphone technology.	cluster‐RCT	**Setting:** School‐based **Provider:** Study staff	**intervention:** 8 months **Follow up:** 18 months	**Population:** Normal (61%) and at risk for OWOB sample *N*: 361 boys **Mean age:** 12.7 years (*SD* 0.5) **Education level:** High schools **SES measure:** School‐level SES, i.e., based on ICSEA. **Ethnicity:** 80.6% Australian 12.2% European 0.6% African 2.2% Asian 4.4% Other	Education/behavioural **Theory:** Social cognitive theory Self‐determination theory	**Intervention:** Teach professional development parents and parental newsletters Students research‐led seminars Enhanced school sports sessions Lunchtime physical activity‐mentoring sessions Smartphone app and Website **Control (waitlist):** Receives intervention after intervention period ends	1.1, 1.2, 1.4, 1.8, 2.2, 2.3, 3.2, 4.1, 5.1, 6.1, 8.7, 10.3, 10.11, 13.1	**SSBs:** Type SSB NR **Subjective:** Validated dietary/physical activity questionnaire	Significant between‐group reduction in SSB intake.
Smith et al. 2014^45^ USA	To evaluate the efficacy of a school‐based intervention on SSB consumption among Appalachian high school students.	Quasi‐experimental (pre–post with no control group)	**Setting:** School‐based **Provider:** Students	**intervention:** 4 weeks **Follow up:** 1‐month follow up	**Population:** Healthy adolescents *N*: 73 boys, 113 girls **Mean age:** 15.8 years (*SD* 1.8) **Education level:** 9–12 graders, high schools **SES measure:** Household‐income based on NSLP‐eligibility (40% NSLP‐eligible) **Ethnicity:** 94.6% White/Caucasian 3% Black/African–American 0.5% Native American 0.5% Asian American 1% > Other	Education/behavioural **Theory:** NR	**Intervention:** Educational programme on media messaging, media coverage of SSBs, written information for school newsletters and local newspapers about SSBs **Control:** No control group	5.1, 8.2, 10.11	**SSBs:** Pop/soda sweetened tea, sweetened coffee drinks, fruit drinks (excluding 100% juice), sports drinks, and energy drinks **Subjective:** Survey	Significant within‐group reduction in SSB intake.
Spook et al. 2016^46^ The Netherlands	To pilot the effects of balance IT, a self‐regulation game on dietary intake and PA among secondary vocational students.	cluster‐RCT (pre–post with a control group)	**Setting:** School‐based **Provider:** Game	**intervention:** 4 weeks	**Population**: Normal (75%) and at risk for OWOB sample *N*: 39 boys, 66 girls **Mean age:** 16.7 years (*SD* 1.10) **Education level:** Lower vocational secondary schools **SES measure:** NR **Ethnicity:** 27% non‐Dutch	Education/behavioural **Theory:** Self‐regulation theory Intervention mapping	**Intervention:** Tailored educational game in which participants are asked to set graded tasks. Users are asked to monitor and evaluate their goals on a daily/weekly basis. Each day, users are prompted with their goals. Visual feedback on self‐reported goal attainment is provided for each goal set. Users are also asked to formulate implementation intentions (these implementation intentions can be set as reminder prompts). Social support is provided through the Balance IT Forum. **Control (waitlist)** Receives the intervention after intervention period ends	1.1, 1.2, 1.4, 2.2, 2.3, 3.2, 7.1, 10.3	**SSBs:** Soft drinks **Subjective:** Validated FFQ	No significant between‐group reduction in SSB intake.
VanEpps et al. 2016^54^ USA	To test the extent to which warning labels for SSBs can influence adolescents' beliefs and hypothetical choices	RCT	**Setting:** Lab‐based **Provider:** Web‐based	**intervention:** ? **Follow up:** ?	**Population:** Healthy adolescents *N*: 1,094 boys, 1,108 girls **Mean age:** 15 years **Education level:** 5th–12th graders, middle‐high schools **SES measure:** Family‐level SES based on parental education level (9.6% with maternal education <high school degree, 6.5% participants with paternal education <high school degree). **Ethnicity:** 31.6% Hispanic 62.9% White 33.6% Black 1.8% Asian 2.1% Native American 0.3% Hawaiian 4.5% Other	Educational/behavioural **Theory:** NR	**Intervention:** A hypothetical vending machine setting in which users receive 6 warning conditions (e.g., SAFETY WARNING: Drinking beverages with added sugar(s) contributes to obesity, diabetes, and tooth decay) **Control (inactive):** Receives no intervention	no BCTs	**SSB definition:** Type SSB NR **Subjective:** Survey	Significant reduction in SSB intake.
Nassau et al. 2014^47^ The Netherlands	To evaluate the impact of the DOiT‐implementation programme on adolescents' adiposity and energy balance‐related behaviours.	cluster‐RCT	**Setting:** School‐based **Provider:** Study staff	**Follow up:** 20‐month follow up	**Population:** Normal (72%) and at risk for OWOB sample *N*: 428 boys, 483 females **Mean age:** 12.8 years **Education level:** prevocational secondary schools **SES measure:** Individual‐level SES based on education level **Ethnicity:** Author mentions including a diverse ethnic sample but participants backgrounds NR	Education/behavioural/environmental **Theory:** Intervention Mapping	**Intervention** Educational programme covering 11 lessons on biology and physical education environmental change options such as encouraging schools to offer additional physical education classes and advice for schools on changes in and around school cafeterias. **Control (usual):** Receives regular curriculum)	1.2, 1.4, 2.2, 2.3, 3.2, 4.1, 5.1, 6.2, 7.1, 7.3, 8.1, 8.7, 10.3, 12.1, 12.5	**SSB definition:** Type SSB NR **Subjective:** Validated dietary questionnaire	Significant within‐group reduction in SSB intake.
Whittemore et al. ^48^ 2012 USA	To compare the effectiveness of two school‐based internet obesity prevention programmes on dietary and physical activity behaviours	cluster‐RCT	**Setting:** School‐based **Provider:** Web‐based	**intervention:** 3 months **Follow up:** 6 months follow up	**Population:** Normal (61%) and at risk for OWOB sample *N*: 77 boys, 130 girls **Mean age:** 15.2 years (*SD* 0.69) **Education level:** High schools **SES measure:** Household‐level SES based on parental income (42% with an income <40.000) **Ethnicity:** 37% White 21.6% Hispanic 28.9% African–American 12.3% Other	Education/behavioural **Theory:** Theory of interactive technology Social learning theory	**Intervention:** School‐based web educational programme composed of two components: HealtheTeen: Students receive lessons on goal‐setting, self‐monitoring, health coaching and social networking. HealtheTeen + CST: Includes similar lessons as HealtheTeen but also lessons on coping skills training, social problem, solving, stress reduction, assertive communication and conflict solving. **Control (usual)** Receives regular curriculum	1.1, 1.2, 2.2, 2.3, 3.2, 5.1, 6.1, 8.1, 9.1, 11.2	**SSB definition:** Soda and fruit juice **Subjective:** Validated dietary questionnaire	Significant between‐group reduction in SSB intake.
Winett et al, 1999^49^ USA	To test the efficacy of the programme with multiple groups of 9th‐ and l0th‐grade girls on reducing calories from SSB.	Quasi‐experimental (pre–post with a control group)	**Setting:** School‐based **Provider:** School staff	**intervention:** ?	**Population:** Healthy adolescents *N*: 103 girls **Mean age:** 14.9 years **Education level:** 9th and 10th graders, high school **SES measure:** Area‐level SES, schools located in low‐SES neighbourhoods **Ethnicity:** NR	Education/behavioural **Theory:** Social cognitive theory	**Intervention:** School‐based educational programme which focuses on dietary and physical activity behaviours. **Control (usual):** Receives regular curriculum	1.1, 1.5, 2.2, 2.3, 5.1, 6.3	**SSB definition:** Soda **Subjective:** 24‐h recall and validated FFQ	Significant between‐group reduction in SSB intake.

Abbreviations: FQs, food frequency questionnaires; NR, not reported; NSLP, national school free/reduced lunch programme; OWOB, overweight/obese, SEIFA, socioeconomic index for areas; SES, socioeconomic status; SSB, sugar‐sweetened beverage; RCTs, randomized controlled trials; WHO, World Health Organization.

Thirteen studies were conducted in the United States,^33^, ^35^, ^36^, ^39^, ^40^, ^41^, ^42^, ^45^, ^48^, ^49^, ^52^, ^53^, ^54^ three in Australia,^34^, ^44^, ^51^ two in Belgium^37^, ^38^ and four in the Netherlands.^43^, ^46^, ^47^, ^50^ Fifteen studies used RCT designs, and seven studies used pre–post designs. Seventeen studies were school‐based interventions,^33^, ^34^, ^35^, ^36^, ^37^, ^40^, ^41^, ^42^, ^43^, ^44^, ^45^, ^46^, ^47^, ^48^, ^49^, ^50^, ^51^ one study was school and football‐based intervention,^38^ two store‐based,^52^, ^53^ one home‐based^39^ and one lab‐based.^54^ The total number of intervention arms was 33 with the majority of studies being one‐arm interventions (*n* = 18; range 1–6 arms). Intervention duration ranged from 2 weeks to 9 months and follow up from 1–20 months. Studies were classified as educational/behavioral (e.g., nutritional education on SSB), environmental (e.g., changes in the physical context) or both. Fifteen studies were classified as educational/behavioral interventions,^34^, ^35^, ^36^, ^38^, ^40^, ^41^, ^42^, ^44^, ^45^, ^46^, ^48^, ^49^, ^50^, ^51^, ^54^ two as environmental^52^, ^53^ and five as both.^33^, ^37^, ^39^, ^43^, ^47^ Seventeen interventions were theory‐driven, of which six were based on one behavioral theory^34^, ^38^, ^40^, ^43^, ^49^ and 11 based on a combination of behavioral theories.^33^, ^35^, ^36^, ^37^, ^41^, ^42^, ^44^, ^46^, ^48^, ^50^, ^51^ Five studies did not use any behavioral theories.^39^, ^45^, ^52^, ^53^, ^54^ Common behavioral theories were social cognitive theory (*n* = 8) and theory of planned behavior (*n* = 5). Two studies measures SSB intake objectively (sales data),^52^, ^53^ and 20 studies assessed SSB subjectively using validated food frequency questionnaires (FFQs), food surveys, dietary questionnaires and 24‐h recalls.^33^, ^34^, ^35^, ^36^, ^37^, ^38^, ^39^, ^40^, ^41^, ^42^, ^43^, ^44^, ^45^, ^46^, ^47^, ^48^, ^49^, ^50^, ^51^, ^54^

Fourteen studies^33^, ^34^, ^35^, ^36^, ^37^, ^38^, ^40^, ^42^, ^43^, ^45^, ^48^, ^49^, ^52^, ^53^ reported type of beverages included in its definition of SSBs.^4^ Eight studies did not report the type of beverages included but reported only measuring SSBs.^36^, ^39^, ^41^, ^44^, ^47^, ^50^, ^51^, ^54^ Majority of the studies had a general SSB category without specifying the impact of the intervention per SSB type. Most of studies only measured whether or not the intervention was effective in reducing SSB consumption.

In most studies, the intervention was compared with a treatment as usual control (*n* = 7), waitlist control (*n* = 4), active control (*n* = 3), inactive control (*n* = 3) or no control (*n* = 5).

### 3.2 Risk of bias of included studies

**FIGURE 2:**
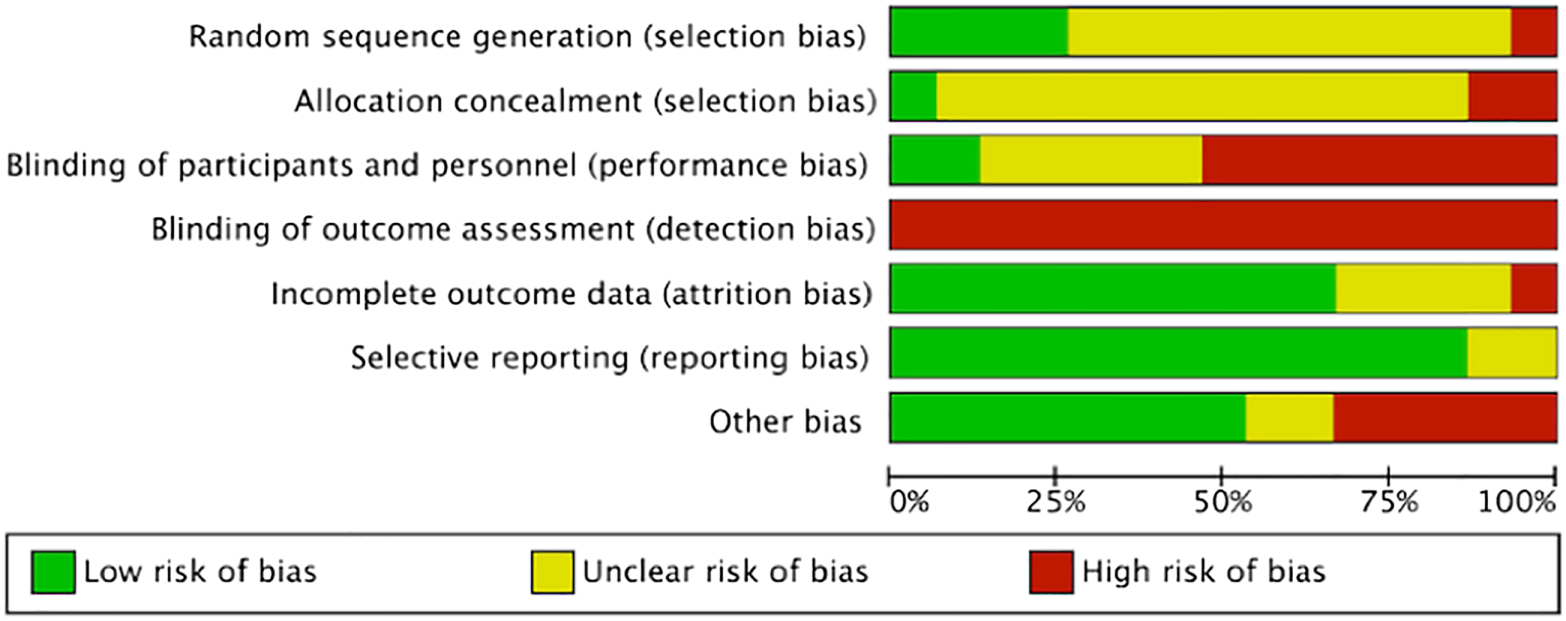
The overall risk of bias assessment of included randomized controlled trials (RCTs)

**FIGURE 3:**
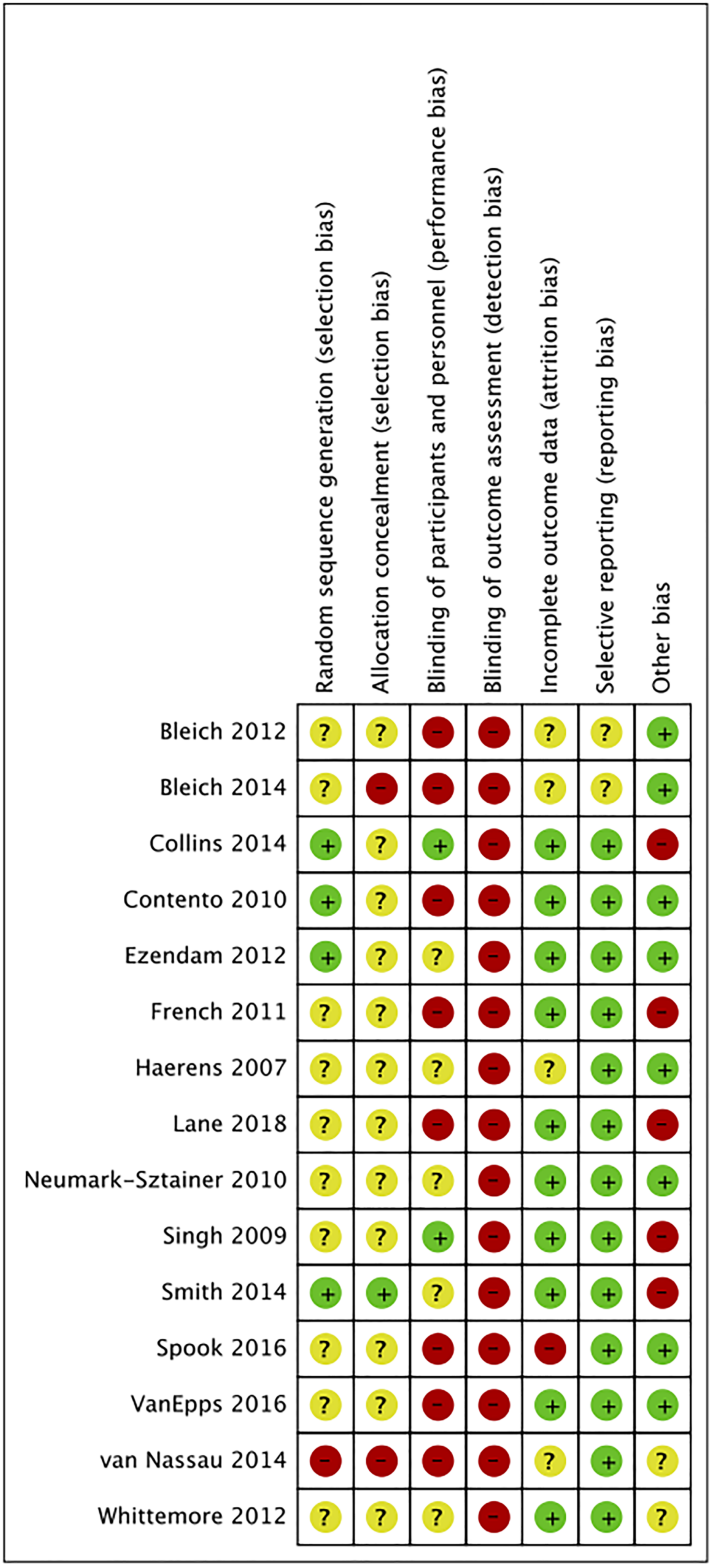
Risk of bias summaries for randomized controlled trials (RCTs)

The risk of bias of RCTs is summarized in Figures 2 and 3. There was a low risk of bias for attrition bias (*n* = 10, 67%), reporting bias (*n* = 13, 87%) and other bias (*n* = 8, 53%). However, for most studies, the risk associated with blinding of assessors (*n* = 15, 100%) was high. In addition, majority of studies were deemed to have an unclear risk of bias due to insufficient descriptions related to randomization (*n* = 10, 67%), allocation concealment (*n* = 12, 80%) and blinding of participants and research staff (*n* = 5, 33%). Overall, the risk of the included RCTs was deemed moderate to high risk of bias.

**FIGURE 4:**
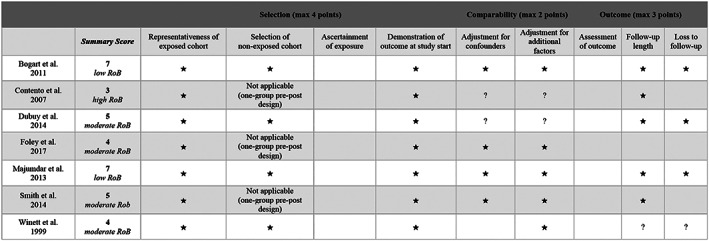
Quality assessment of nonrandomized controlled trials based on the Newcastle–Ottawa quality tool

The risk of bias of non‐RCTs was evaluated using the Newcastle–Ottawa tool and is presented in Figure 4. Four studies were judged to have a ‘moderate’ risk of bias, two were rated as having a ‘low’ risk of bias and one ‘high’ risk of bias.

### 3.3 Meta‐analysis: Effectiveness of interventions on reducing SSB intake

**FIGURE 5:**
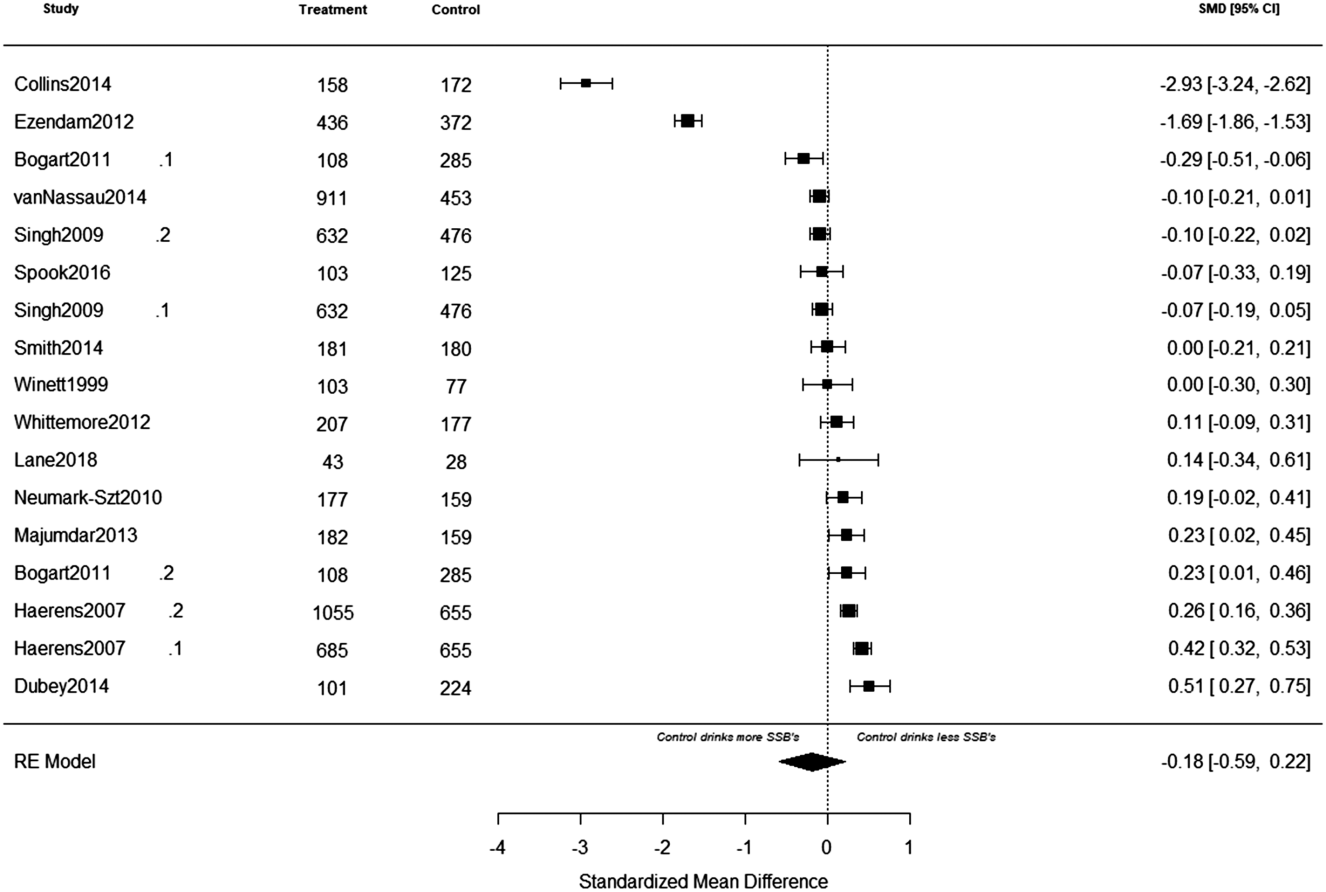
A summary of individual study effects at pre‐intervention. The comparison between intervention and control groups at pre‐intervention are derived from separate random‐effects models. Hedges' *g* point estimates are represented by filled squares, and the filled diamond represents the estimated summary effect sizes (standardized mean difference). Error bars and diamond width represents the 95% confidence intervals. Positive values represent favouring the intervention whereas negative values represent favouring the control groups

**FIGURE 6:**
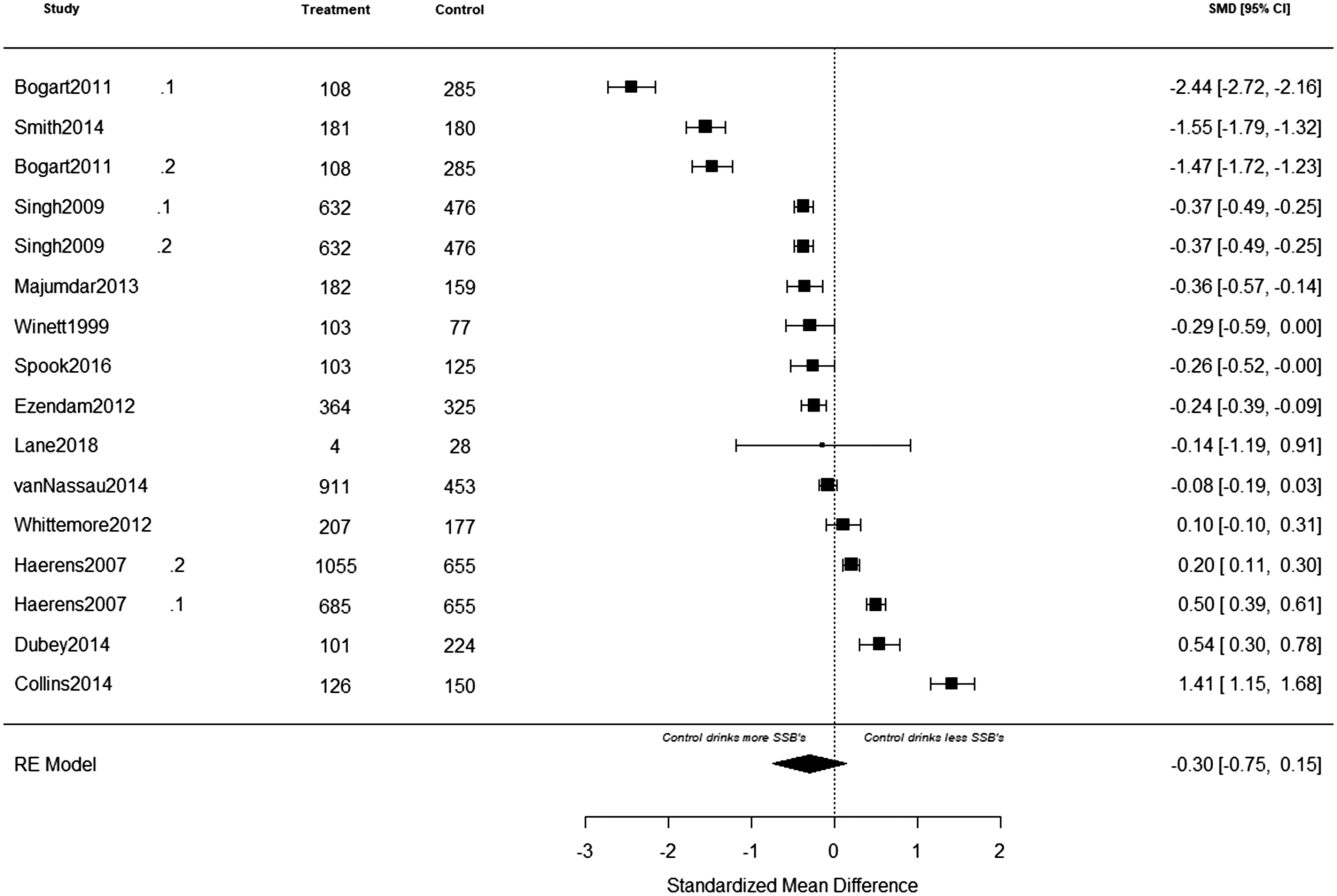
A summary of individual study effects at post‐intervention. The comparison between intervention and control groups at pre‐intervention are derived from separate random‐effects models. Hedges' *g* point estimates are represented by filled squares, and the filled diamond represents the estimated summary effect sizes (standardized mean difference). Error bars and diamond width represents the 95% confidence intervals. Positive values represent favouring the intervention whereas negative values represent favouring the control groups

Forest plots of individual study effects at pre‐ and at post‐intervention are presented in Figures 5 and 6, respectively. Cluster‐robust models revealed no significant change in SSB intake between groups at pre‐intervention (*g* = −0.24, 95% CI [−0.80, 0.32], *p* = 0.36), and at post‐intervention (*g* = −0.23, 95% CI [−0.76,0.29], *p* = 0.35). In addition, we observed no significant change in SSB intake between groups from pre‐ to post‐intervention (*F* (1, 13) = 0.00, *p* = 0.99). At pre‐intervention, results of the multilevel models showed no significant change in SSB intake between groups (*g* = −0.18, 95% CI [−0·58, 0·22], *p* = 0.37). Heterogeneity was substantial (*Q* (*df* = 16), 889.73, *I* ^2^ = 99%). Likewise, at post‐intervention, there was no significant change in SSB intake between groups (*g* = −0.30, [95% CI [−0.75, 0.14], *p* = 0.19), and heterogeneity was substantial (*Q* (*df* = 15), 898.27, *I* ^2^ = 99%). Inspection of the forest plots (Figures 5 and 6) revealed three studies that were identified as outliers.^33^, ^34^, ^50^ To check if results would change after removing the three influential studies, we reran the primary meta‐analysis. Results of the multilevel models showed no significant change in SSB intake between groups at pre‐intervention (*g* = 0.12, 95% CI [−0.02, 0.25], *p* = 0.08), at post‐intervention (*g* = −0.31, 95% CI [0.78, 0.15], *p* = 0.16), and from pre‐ to post‐intervention (*F* (1, 11) = 4.77, *p* = 0.06).

Separate random‐effects models were used to assess the effects of potential moderators. No significant moderating effects of study design (RCT vs. non‐RCT), intervention type (educational/behavior vs. environmental), intervention component (single vs. multicomponent) and total number of BCTs were observed. Re‐analysing the primary analyses after excluding influential studies revealed different results. There was a significant moderating effect of intervention type on SSB intake at post‐intervention (QM (*df* = 1) = 7.26, *p* = 0.01), with interventions with an educational/behavioral component showing a reduction in SSB intake (*β* = −0.54, *SE* = 1.76, *p* < 0.01) relative to interventions with an environmental component (*β* = 0.29, *SE* = 0.25, *p* = 0.26).

**FIGURE 7:**
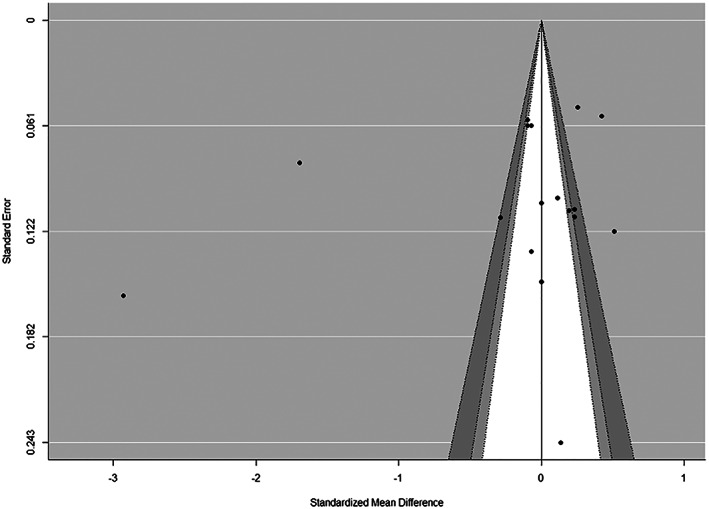
Contour‐enhanced funnel plots for sugar‐sweetened beverage (SSB) intake at pre‐intervention. In these plots, individual study effect estimates are plotted against standard errors to determine whether statistically significant studies are overrepresented in the included studies. The light grey area denotes *p* values between 0.1 and 0.05, the dark grey region captures *p* values between 0.05 and 0.01, and the white region includes *p* values greater than 0.1. Because there is no overrepresentation of studies within the light and dark grey regions, this is suggestive of low/no publication bias

**FIGURE 8:**
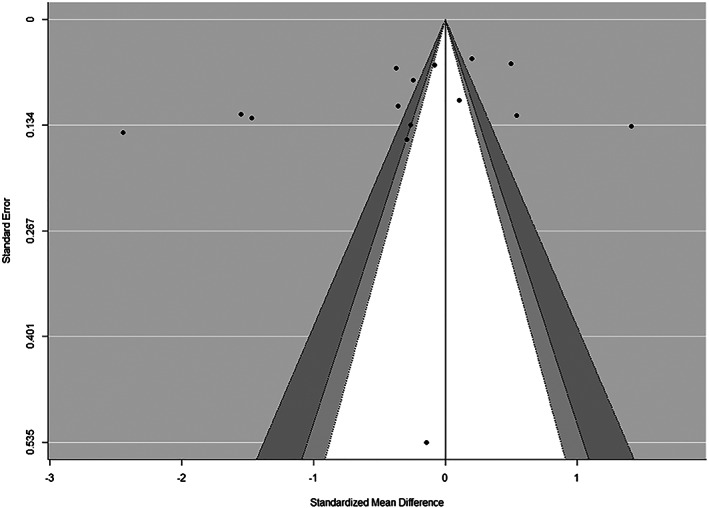
Contour‐enhanced funnel plots for sugar‐sweetened beverage (SSB) intake at post‐intervention. In these plots, individual study effect estimates are plotted against standard errors to determine whether statistically significant studies are overrepresented in the included studies. The light grey area denotes *p* values between 0.1 and 0.05, the dark grey region captures *p* values between 0.05 and 0.01, and the white region includes *p* values greater than 0.1. Because there is no overrepresentation of studies within the light and dark grey regions, this is suggestive of low/no publication bias

Visual inspection of funnel plots (Figures 7 and 8) using the Egger regression asymmetry test suggested no evidence of publication bias to higher values (*p* > 0.05).

### 3.4 Effectiveness of BCTs (narrative synthesis)

Table 3 summarizes the BCTs identified in included studies and applied in the 33 active interventions arms. Of the 93 empirical BCTs, 30 were coded one or more times with a total of 153 BCTs coded across the active intervention arms. Three studies did not use BCTs.^52^, ^53^, ^54^ The median number of BCTs used in all studies was seven (range: 3–15). A median of eight BCTs (range: 7–8) was used in four single‐component interventions.^35^, ^36^, ^40^, ^50^ A median of seven BCTs (range: 3–15) was used in 15 multicomponent interventions.^33^, ^34^, ^37^, ^38^, ^39^, ^41^, ^42^, ^43^, ^44^, ^45^, ^46^, ^47^, ^48^, ^49^, ^51^ The most frequently used BCTs included information on health consequences (*n* = 16), problem‐solving and/or barrier identification (*n* = 13), and goal‐setting of behavior (*n* = 12). Studies classified as educational/behavioral used less BCTs (median = 7, range: 3–14) compared with studies classified as educational/behavior/environmental (median = 10 BCTs, range: 5–15). Seven studies were deemed ‘very effective’,^35^, ^41^, ^43^, ^44^, ^48^, ^49^, ^50^ six ‘quite effective’^33^, ^36^, ^40^, ^45^, ^47^, ^51^ and six ‘non‐effective’.^34^, ^37^, ^38^, ^39^, ^42^, ^46^ ‘Very effective’ studies used more BCTs (mean = 4.0, *SD* = 1.9) compared with ‘non‐effective’ studies (mean = 3.0, *SD* = 1.0) (Table 4).

**TABLE 3:** Frequencies of behavior change techniques used in included studies

Behavior change technique	*N*	%
1.1 Goal setting (behavior)	12	36.4
1.2 Problem solving/barrier identification	13	39.4
1.3 Goal setting (outcome)	1	3.0
1.4 Action planning (including implementation intention)	11	33.3
1.5 Review behavior goals	1	3.0
1.8 Behavioral contract	2	6.1
1.9 Commitment	2	6.1
2.2 Feedback on behavior	9	27.3
2.3 Self‐monitoring of behavior	11	33.3
3.2 Social support (practical)	11	33.3
4.1 Instruction on how to perform the behavior	8	24.2
4.3 Re‐attribution	1	3.0
4.4 Behavioral experiments	1	3.0
5.1 Information about health consequences	16	48.5
5.2 Information about social and environmental consequences	1	3.0
6.1 Modelling/demonstration of the behavior	6	18.2
6.2 Social comparison	5	15.2
6.3 Information about others approval	1	3.0
7.1 Prompts/cues	5	15.2
7.3 Reduce prompts/cues	3	9.1
8.1 Behavioral/practice/rehearsal	4	12.1
8.2 Behavior substitution	1	3.0
8.7 Graded tasks	3	9.1
9.1 Credible source	2	6.1
10.3 Nonspecific reward (includes positive reinforcement)	6	18.2
10.11 Future punishment (includes threat)	4	12.1
11.2 Reduce negative emotions	2	6.1
12.1 Restructuring the physical environment	5	15.2
12.5 Adding objects to the environment	3	9.1
13.1 Identification of self as role model	3	9.1

**TABLE 4:** Frequency of behavior change techniques coded in ‘very effective’, ‘quite effective’ and ‘non‐effective’ studies

Behaviour change technique	Very effective studies (*n* = 7)	Quite effective studies (*n* = 6)	Non‐effective studies (*n* = 6)
1.1 Goal setting (behavior)	6	3	3
1.2 Problem solving/barrier identification	6	3	4
1.4 Action planning (including implementation intention)	5	3	3
2.2 Feedback on behavior	5	2	2
2.3 Self‐monitoring of behavior	6	2	3
3.2 Social support (practical)	4	2	5
4.1 Instruction on how to perform the behavior	4	2	2
5.1 Information about health consequences	7	5	4
6.1 Modelling/demonstration of the behavior	2	2	2
6.2 Social comparison	2	1	2
7.1 Prompts/cues	2	1	2
10.3 Non‐specific reward (includes positive reinforcement)	3	1	2
12.1 Restructuring the physical environment	1	2	2

*Note:* Studies are deemed ‘very effective’ if *between‐group* differences in SSB intake were reported, ‘quite effective’ if *within‐group* changes in SSB intake were reported, *and* ‘non‐effective’ if no *between* nor *within‐group* changes in SSB intake were reported.

**FIGURE 9:**
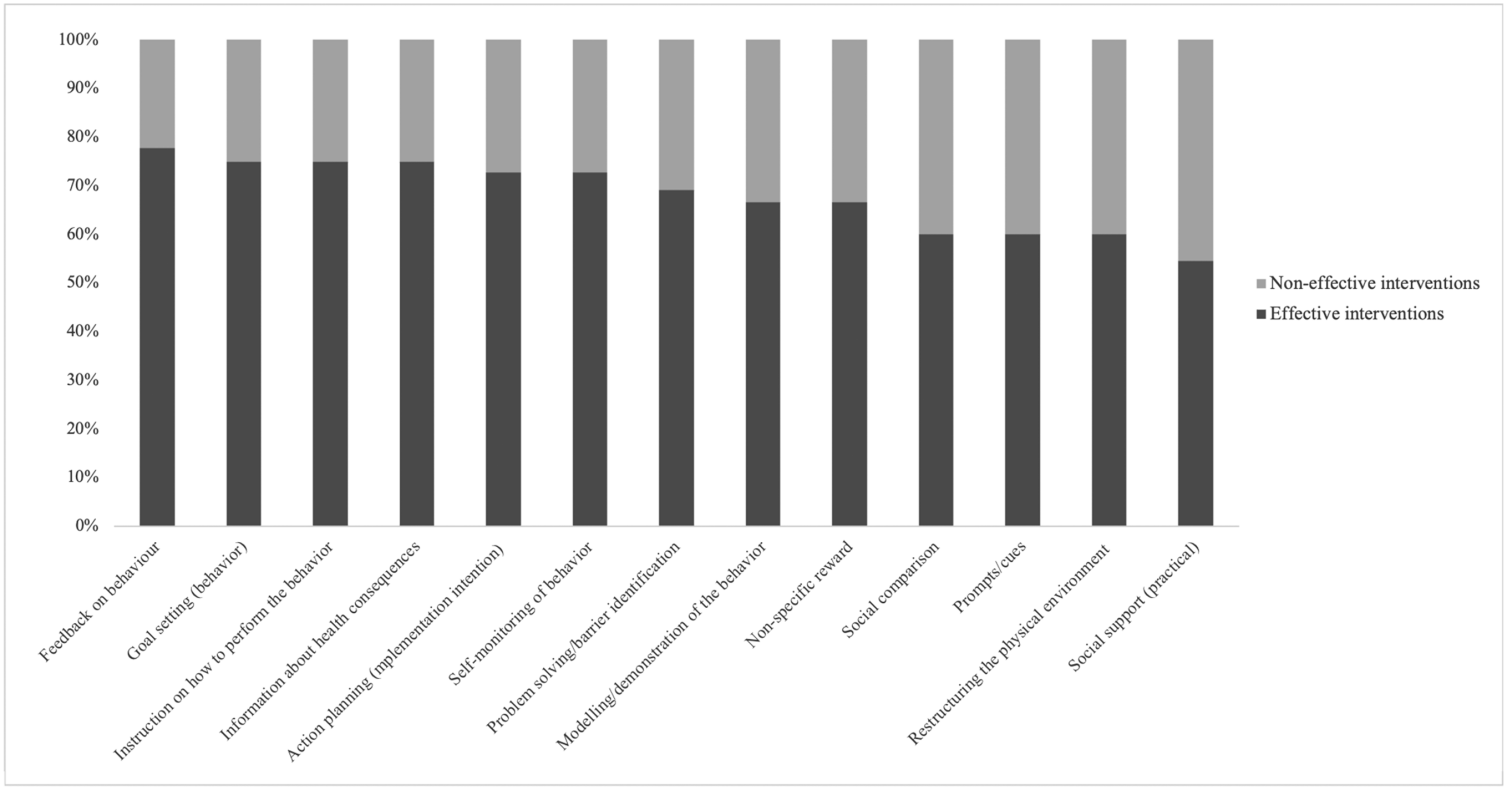
Percentage effectiveness of behavior change techniques (BCTs)

The percentage effectiveness ratio of the BCTs is presented in Figure 9. In total, 13 BCTs had an effectiveness ratio >50%. The BCTs with the highest effectiveness ratio were feedback on behavior (*n* = 8/9, 78%), goal‐setting of behavior (*n* = 9/12, 75%), instruction on how to perform a behavior (*n* = 6/8, 75%), health consequences (*n* = 12/16, 75%), followed by action planning including implementation intention (*n* = 8/11, 73%), self‐monitoring of behavior (*n* = 8/11, 73%) and problem‐solving and/or barrier identification (*n* = 9/13, 69%).

## 4 DISCUSSION

The aims of the current review were twofold: (a) to examine the effectiveness of behavioral interventions aimed at reducing SSB intake among 12‐ to 18‐year socioeconomically disadvantaged ethnic minority adolescents and (b) to identify which BCTs contribute to a reduction in SSB intake in this target group. In total, 22 studies aiming to reduce SSB intake were identified, of which 19 were included in a meta‐analysis. Most importantly, the most effective BCTs identified in the narrative synthesis of this review were feedback on behavior, goal‐setting of behavior, instruction on how to perform the behavior, information about health consequences, action planning including implementation intention, self‐monitoring of behavior, problem‐solving and/or barrier identification and modelling/demonstration of the behavior.

Overall, our meta‐analysis showed that behavioral interventions were not effective in reducing SSB intake, which is in line with another systematic review and meta‐analysis.^27^ In their meta‐analysis, Champion et al.^27^ included four studies and found no evidence found no evidence of effectiveness of interventions on reducing SSB intake. However, in their review, Champion et al.^27^ included adolescents from the general population, used only one SSB measure, and included only school‐based eHealth interventions. Further, in contrast to our meta‐analysis, the meta‐analyses of two previous reviews^4^, ^28^ reported that behavioral interventions lead to a small‐sized reduction in SSB intake, but with considerable heterogeneity.

There are several possible explanations for the mixed findings. First, previous reviews^4^, ^27^, ^28^ included adolescents from the general population, whereas we primarily included disadvantaged minority adolescents, given that SSB intake is more common in these groups than in the general adolescent population.^3^ Evidence suggests that *one‐size‐fits‐all* interventions that are delivered in the same way to advantaged and disadvantaged groups may unintentionally lead to *intervention‐generated inequalities*.^22^ White et al.^65^ state that resultant inequalities can be generated at multiple points throughout the intervention process. For instance, compliance with behavioral interventions may be lower among underserved groups because they lack resources (e.g., finances and knowledge), are less health‐conscious and have less supportive social and physical environments. The corollary is that interventions that are tailored to the needs of these groups will more likely be effective to change dietary behaviors. Moreover, underserved adolescents are often underrepresented in intervention studies because they are difficult to recruit and generally often hard to reach. A possible reason for why these groups may not engage in intervention studies is because they are hindered by pressing daily struggles. Daily struggles such as coming from poor households, residing in unstable social and physical environments, perceiving discrimination, and dealing with financial scarcity can impose burdens on the mind, leaving less ‘mental’ room which in turn can deflect the adolescents' interests to change their unhealthy dietary behaviors.^66^ Second, another factor that can explain the observed difference in findings is possibly related to the heterogeneity in the measures of SSBs used. For example, previous reviews^4^, ^27^, ^28^ only included studies that reported volumetric measures of SSB intake (e.g., ml of SSBs consumed per day/week). In contrast to these reviews, given that there is no consensus on a clear definition of SSB intake, we did not set any criteria on how individual consumption of SSB needed to be reported in the articles. For the current meta‐analysis, we considered frequency measures of SSB intake (e.g., SSB consumed per day) and proportion of individuals who reported consuming a given quantity of SSB. Moreover, given the definition of SSBs, which includes a host of drinks such as soda, diet drinks, flavoured juice drinks, sports drinks and sweetened coffee and tea,^4^ we also included effect estimates reported either individually for each type of SSB (e.g., soft drinks and energy drinks) or collectively (e.g., all types of SSB grouped into one category). Finally, in contrast to the review by Champion et al.,^27^ we included behavioral interventions implemented at the household, neighborhood, school, store and community levels.

In the narrative review, we compared individual BCTs in effective and non‐effective interventions to identify BCTs that were associated with intervention effectiveness. Thirteen BCTs had an effectiveness ratio >50% of which five included the self‐regulatory BCTs derived from the control theory.^67^ These included feedback on behavior (*n* = 8/9, 78%), goal‐setting of behavior (*n* = 9/12, 75%), action planning including implementation intention (*n* = 8/11, *n* = 73%), self‐monitoring of behavior (*n* = 8/11, 73%) and problem solving and/or barrier identification (*n* = 9/13, 69%). An example of the BCT feedback on behavior, is to provide tailored feedback on whether or not participants met their goal of drinking less SSBs per week, to provide evaluative feedback on participants progress of drinking less SSBs per week or providing informative feedback with strategies on how to overcome barriers to reduce SSB intake. Examples of the BCTs goal‐setting, action planning including implementation intention, self‐monitoring and problem‐solving and/or barrier identification are (a) to specify and set a goal to reduce individual SSB intake by one serving per day or per week; (b) to make a plan and specify *when* and *where* one would consume less SSBs; (c) to record and monitor one's SSB intake; and (d) to provide situations where consuming less SSBs would be challenging and prompt participants to identify solutions and strategies to overcome barriers preventing them from drinking less SSBs. These results are comparable with previous reviews and meta‐analyses, which found that self‐regulatory BCTs were associated with better dietary and physical activity behavior.^29^, ^63^, ^68^, ^69^, ^70^, ^71^ Further, the BCTs instruction on how to perform a behavior (*n* = 6/8, 75%) and information about health consequences (*n* = 12/16, 75%) were also found to be effective. An example of the BCT provision of instruction is to provide participants specific instructions on how they can reduce their SSB intake. The effectiveness of the BCT providing instruction is similar to another review supporting the efficacy of providing instructions for improving dietary outcomes in adults.^72^ The BCT health consequences can be used, for example, to provide factual information on health consequences related to prolonged intake of SSBs. In a comparable review, this BCT was commonly used in educational/behavioral school‐based interventions.^29^

This review has several strengths. To the best of our knowledge, no review to date has examined the effectiveness of behavioral interventions targeting SSB intake in socioeconomically disadvantaged ethnic minority adolescents. Further, the novelty of this review is that we are the first to explore which BCTs are associated with effective interventions in this diverse sample of adolescents. This is another strength, just as the robust method applied to code the BCTs, using the most comprehensive taxonomy available.

Although the findings of this review and meta‐analysis add new knowledge to the scientific literature, there are several limitations that need to be addressed. First, our meta‐analysis was based on a small number of studies, and significant heterogeneity was present between the studies. Second, high or unclear risk existed regarding blinding of assessors, randomization allocation and concealment of participants/research staff, and overall, we deemed the risk of bias to be moderate to high for RCTs and low to moderate for non‐RCTs. Third, given the insufficient quantity of studies, it was not possible to conduct proper moderator analyses. Instead, we conducted exploratory analyses to assess the impact of potential moderators. However, due to the considerable amount of heterogeneity observed, the findings of these analyses should be interpreted with caution. Fourth, given that different BCTs and theoretical frameworks were used in included studies, it was not possible to examine, for instance, which specific BCTs and BCT combinations were effective in reducing SSB intake. Fifth, examining the long‐term maintenance of the effects of behavioral interventions on SSB intake was not possible in the meta‐analysis because most studies had short follow up. Sixth, coding of BCTs was problematic due to inconsistencies in reporting of BCTs. In this review, a stringent coding strategy was used. A BCT was coded as *present*, only if it was sufficiently described or the authors referred to a BCT taxonomy. The problematic issue related to coding of BCTs has been identified in previous reviews.^63^, ^64^ Further, there was an overrepresentation of studies conducted in the USA, which limits generalizability to other contexts (e.g., built environment) and continents (e.g., differences in cultural and migration history of minorities in Europe). Finally, only studies reported in English were reviewed, meaning eligible studies in other languages may have been missed.

This review highlights important directions for future research. First, more high‐quality studies examining the effectiveness of behavioral interventions among disadvantaged minority adolescents are warranted. Second, future research should consider the unique conditions and background factors (self‐efficacy, cultural values and norms) that characterize this target group when designing interventions for them. Third, given that a large part of the heterogeneity remains unexplained, well‐powered future studies are needed to conduct proper subgroup analyses to identify potential moderators influencing intervention effectiveness. Fourth, because it was not possible to identify which specific BCTs or BCT combinations were effective in changing SSB behavior, future investigation is warranted. Moreover, although 77% of the included studies reported using a theoretical framework, there was inconsistent and incomplete information on how different theories were operationalized in intervention design and implementation of the included studies. Thus, future work should be transparent and provide complete information on how specific types of theory/theories are used in intervention design and implementation of behavioral interventions targeting underserved adolescents. Fifth, given that long‐term effects of included behavioral interventions could not be explored, additional research is needed to examine whether changes in SSB behavior are sustained over time. Sixth, to help identify active BCTs, future research should provide a checklist of BCTs used in their studies as supplementary materials. This will not only ease the process of identifying active BCTs but will also aid in the development of possibly effective and replicable behavioral interventions.

Further, this review demonstrates several BCTs in reducing SSB intake in underserved adolescents. Based on the narrative synthesis, individual BCTs with greater effectiveness were those that targeted participants' self‐regulatory skills such as feedback on behavior, goal‐setting, self‐monitoring, action planning including implementation intention and problem‐solving/barrier identification. Overall, these findings suggest that providing individuals personalized feedback which they can translate into behavior change as well as enhancing their self‐regulatory capacity to make them more autonomous about their own behaviors may be more effective rather than offering them generic behavioral interventions. Therefore, in order to effectively facilitate behavior change, researchers should consider incorporating skill‐building and self‐regulatory BCTs not only to curb SSB intake but also to avoid unintended consequences (i.e., increased intake of sports drinks or energy drinks). Possible ways to curb SSB intake and avoid unintended consequences could be, for example, to promote consumption of tap water, provide tap water access (installing bottle fillers/fountain drinking water units) or to provide bottled water coolers installed within schools. Finally, given the heterogeneous nature of the included studies, studies reported changes in SSB intake differently. For example, some studies reported changes in one or more than one type of SSBs, had unclear totals for SSBs or had unclear definition of SSBs. Thus, it is plausible, for example, that while frequency of SSB intake reduced, volumetric measures of SSBs or an intake in other SSBs (e.g., sport drinks and energy drinks that are popular among adolescents) increased, which were not measured or reported. Therefore, given the lack of consensus on how to measure individual SSB intake, it is important that efforts are made to establish a clear definition of SSB. In addition, given that different types of SSBs exists, authors are encouraged to include all types of SSBs that meet the definition of SSB and report the impact of an intervention on each type of SSB separately as this will facilitate a better understanding of the kind of effect behavioral interventions would have on the different types of SSBs.

In sum, the findings of the review indicate that behavioral interventions that may be effective for the general adolescent population may not be effective for socioeconomically disadvantaged ethnic minority adolescents. Thus, further high quality‐research is needed to develop behavioral interventions tailored to the needs of this target group to effectively reduce SSB intake. Further, our findings demonstrate that self‐regulatory BCTs congruent with the control theory may be effective components for reducing SSB intake in underserved adolescents. However, given the lack of studies including each BCT, this review could not identify active BCTs associated with intervention effectiveness. Thus, more research is needed before confirming which BCTs can be considered having a greater effect than others.

## CONFLICT OF INTEREST STATEMENT

The authors have no relevant interests to declare.

## AUTHOR CONTRIBUTION

S. S. and E. G. contributed to the original idea and research questions to be reviewed. S. S. and E. G. wrote the initial draft of the manuscript. S. S. was involved in data extraction, coding and interpretation of BCTs, determined methodological quality of included studies, analysed the data and contributed to the subsequent drafts of the manuscript. J. J. was involved in all statistical analyses. A. S., G. N., J. W. and S. D. provided essential guidance and supervision at all stages of the review. All authors have read and approved the final manuscript.
